# Study on Optimization Technology to Strengthen Ni-Based Composite Coating Electroplate Containing Nanodiamond

**DOI:** 10.3390/ma12101654

**Published:** 2019-05-21

**Authors:** Meihua Liu, Dongai Wang, Huaiwen Wang, Yan Shi, Bing Liu, Feihui Li, Yunlan Gong, Wengang Zhang

**Affiliations:** 1 School of Mechanical Engineering, Tianjin University of Commerce, Tianjin 300134, China; lmhua2007bd@126.com (M.L.); wangda@tjcu.edu.cn (D.W.); shyan@tjcu.edu.cn (Y.S.); liubing@tjcu.edu.cn (B.L.); 2 Department of Applied Chemistry, Tianjin University of Commerce, Tianjin 300134, China; tjlifeihui@tjcu.edu.cn (F.L.); tjcugyl@126.com (Y.G.); 3 Tianjin Chanyu Superhard Sci-Tech Co. Ltd., Tianjin 300384, China; cnchanyu@163.com

**Keywords:** Ni-based composite coating, nanodiamond, polarization behavior, electroplate process, optimization technology

## Abstract

Ni-based composite coating containing nanodiamonds was deposited on the substrate of Q235A low-carbon steel in a traditional Watts solution, without any additive. The nanodiamond grains prepared by detonation synthesis were measured by Transmission electron microscope (TEM) and X-ray diffraction (XRD). The electrochemical behavior of Ni^2+^ ion in the composite bath including nanodiamonds was studied by linear sweep voltammetry experiments, and the morphology, elastic modulus, and hardness of Ni-based composite coating were characterized using Scanning Electron microscope (SEM) and the nano-indenter XP tester. Effects of the nanodiamond concentration in the bath, stirring speed, and the electroplate mode on the properties of Ni-based composite coating were investigated. The results show that the reduction of Ni^2+^ ion in the electroplating process increased initially, and then decreased as the nanodiamond concentration in the bath increased from 4 g/L to 16 g/L, irrespective of whether direct current (DC), single-pulse, or double-pulse electroplating mode was used. The highest over-potential could be obtained when the nanodiamond concentration in the bath was 8 g/L. Moreover, the hardness and elastic modulus of the composite coating prepared by the DC electroplating mode were 4.68 and 194.30 GPa, respectively. By using the same plating parameters, the coating prepared by the double-pulse electroplating mode showed better properties, with hardness and elastic modulus values of 5.22 and 197.38 GPa, respectively.

## 1. Introduction

The development of composite coatings has made great progress so far, and composite coatings have been applied in many fields. Studies of composite coatings in the early stages were on the microscale level. With the development of nanotechnology, research of composite coatings has been developed for the nanoscale level. It has been found that nanocomposite coatings have much better hardness, abrasion resistance, and friction reduction as compared to conventional composite coatings [1,2,3,4,5]. Grosjean et al. prepared Ni-SiC composite coatings on copper and gold substrate, and studied the effects of nano-SiC particle content on the microhardness and abrasion resistance of the coatings [6]. The researchers found that the hardness of the coating was enhanced, with an increased number of SiC particles in the coating, and reached a highest value of 1300 HV. Hamed et al. studied the properties of composite coating prepared by nanodiamonds without any surfactants, and found that the coating had higher corrosion resistance and hardness [7]. Jappes studied electroless composite nickel-plating of polycrystalline diamond, and found that the abrasion resistance of the coating after heat treatment increased significantly [8]. Lee investigated the effects of the electroplating method on the performance of nickel/nanodiamond composite coatings deposited on Cu alloy. The microhardness value of the specimen reached up to 611 HV [9]. Luan, Petrov studied nickel/nanodiamond composite coating obtained by electroplating technology. The microhardness of Ni/nanodiamond composite coatings was about 460 HV and 430 HV [10,11]. This value is almost three times that without nanometer diamond powder addition. Wang studied Ni/nanodiamond composite coatings codeposited on AISI-1045 steel substrates by conventional electrodeposition methods. The microhardness of Ni/nanodiamond composite coatings was about 700 HV [12]. Extensive research on nanodiamond composite electroplating technology began in the 1990s, followed by studies on nanodiamond applications in the field of composite coating [13,14,15,16,17,18]. All of the previous studies drew a consistent conclusion: That the nanodiamond composite coating had good friction performance, and thus very good application prospects.

Although a lot of progress has been made on nanodiamond composite coatings, the research available in this area is still limited in terms of depth and breadth. In order to provide technical guidance for the preparation of high-strength Ni/nanocomposite coatings, we explored the optimum formulation and process parameters for electroplating of Ni/nanodiamond composite in this paper. For this purpose, a low-carbon steel, Q235A, was chosen as the substrate and linear sweep voltammetry was used to test the electrochemical behavior of Ni^2+^ on the Q235A substrate in Watts nickel-plating solution, with different nanodiamond contents and at different stirring speeds. The effects of nanodiamond content and stirring speed on the properties of Ni/nanodiamond composite coatings—such as hardness, elastic modulus, surface roughness, and so on—were investigated, based on which, the most suitable electroplating parameters were selected. Finally, different electroplating modes—direct current (DC) electroplating, single-pulse, and pulse electroplating—were carried out and compared.

## 2. Experimental

### 2.1. Substrate Specifications and Pretreatment

**Figure 1:**
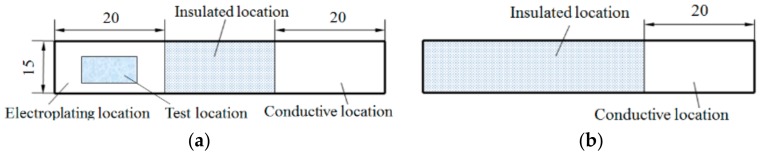
Q235A substrate specification diagram: (**a**) Electroplated surface, and (**b**) nonelectroplated surface.

Q235A mild steel with a size of 60 mm × 15 mm × 2 mm was chosen as the substrate. The electroplating area was sealed by tape, with dimensions of 20 mm × 15 mm × 3 mm and an area of 30 mm^2^, as shown in Figure 1. The dark part in the middle of the electroplating region in Figure 1 is the measurement region, with an area of 10 mm × 5 mm.

**Figure 2:**
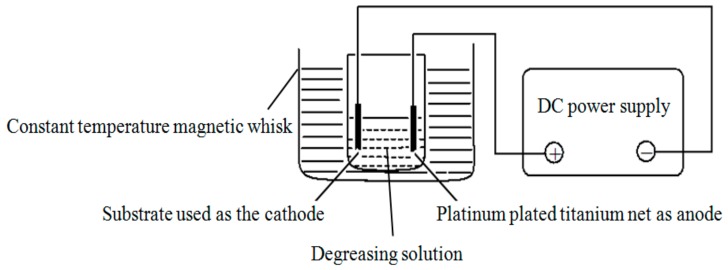
Electrochemical degreasing device.

Pretreatment was conducted on the substrate after sealing. In brief, mechanical polishing was first carried out on the substrate. After that, 60#, 80#, and 120# corundum wheels were used successively to complete the grind step. Then, soft grinding wheels with particle sizes of 240#, 400#, and 600# were used separately in turn to polish the substrate until the surface had no obvious scratches. After mechanical treatment, chemical degreasing treatment was carried out on the substrate, followed by cathodic electrochemical degreasing treatment, during which the Q235A substrate and a platinum mesh were connected with the negative and positive electrode of the power supply, respectively. The electrochemical degreasing device is shown in Figure 2, and the solution formula and process parameters of the degreasing treatment are listed in Table 1. Finally, in order to ensure a clean surface and enhance the binding force between the coating and the substrate, the electroplating area on the substrate was rinsed with distilled water, immersed in 3% HCl solution at room temperature for 10 s, and then rinsed with distilled water again before measurement and electroplating.

### 2.2. Nanodiamond Purification and Solution Preparation

First, the mixed diamond particles prepared by the detonation synthesis method were dispersed in water along with the dispersant, prepared by Tianjin Chanyu Superhard Sci-Tech Co., Ltd. (Tianjin, China). The diamonds were sufficiently dispersed and processed for 2 h using a powerful ultrasonic cleaner. The separation factor of the centrifuge was 23,669*g* and the centrifugation time was 120 h, where the parameter g was the gravitational acceleration. Large-sized diamond particles were removed by a large-capacity centrifuge, medium-sized diamond particles were removed by vacuum filtration, and nanodiamonds with a grain size of less than 10 nm remained in the liquid.

### 2.3. Composition of Plating Solution and Process Conditions

The composition of the Ni/nanodiamond composite bath was as follows: 240.0 g/L NiSO_4_·6H_2_O, 20.0 g/L NiCl_2_·6H_2_O, 20.0 g/L H_3_BO_3_, 0.5 g/L sodium dodecyl sulfate (SDS), and 4–16.0 g/L nanodiamond. In order to avoid agglomeration of the nanodiamond particles, the nanodiamond solution was ultrasonically treated under a temperature of 45 °C for 1 h before preparation of the bath.

The bath was prepared as follows: First, 500 mL of distilled water was added to a 1 L beaker and heated to 50 °C in a constant temperature water bath. After that, NiSO_4_·6H_2_O, NiCl_2_·6H_2_O, and SDS were successively added to the water. Then another beaker was taken, and 200 mL of distilled water was added to it. The water was heated to 90 °C, followed by the addition of H_3_BO_3_. The above 2 solutions were stirred until the chemicals were completely dissolved. Then the solutions were mixed and cooled down to room temperature. Nanodiamond solution was then added to the mixed solution, according to the desired concentration. Finally, the solution was transferred to a 1 L volumetric flask and made up to constant volume. Prior to use, the pH value of the solution needed to be adjusted to 4, using 10% H_2_SO_4_ solution or 10% NaOH solution.

**Figure 3:**
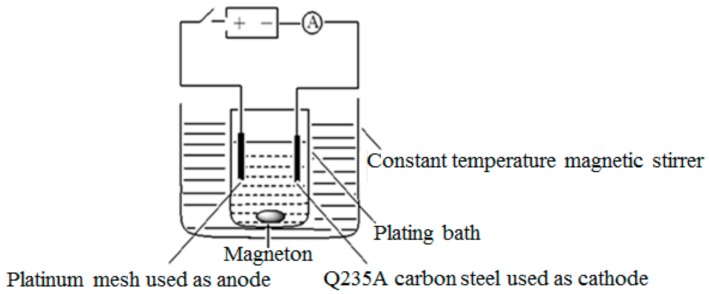
Schematic diagram of the electroplating device.

Based on previous research [19,20], the process parameters of Ni/nanodiamond composite coatings by the DC plating mode were generally within the following ranges: Cathodic current density 1.5 to 5 A/dm^2^, pH value 3.5 to 5.0, temperature 40 to 60 °C, and stirring rate 0 to 100 r/min. The DC electroplating process parameters were selected as follows: Temperature 45 °C, pH value 4, cathodic current density 2 A/dm^2^, and plating time 1 h. The electroplating device is shown in Figure 3.

For the pulse electroplating, an SMD-10 CNC double-pulse power supply was used. Selection of suitable positive and negative pulse parameters was very important, because it directly affects the crystallinity, deposition rate, thickness distribution, and impurity content of the coating. Double-pulse electroplating process parameters include pulse frequency, average current, operating time, duty cycle, and so on. In general, 10–30% is considered appropriate for the duty cycle of the positive or negative pulse, and the pulse frequency is generally selected to be around 1000 Hz in actual applications.

Based on the effect of stirring speed on the electrochemical behavior of the composite plating solution and the test results of properties of the composite coating prepared by DC electroplating, the selected pulse electroplating conditions were as follows: Nanodiamond concentration was 8 g/L, stirring speed was 10 r/min, and other process parameters were unchanged. Studies have shown that increasing the pulse width can result in refined coating crystals [21]. In this experiment, 2 positive pulses with operating times of 50 and 100 ms were selected for the comparison test. The double-pulse electroplating process parameters are shown in Table 2.

### 2.4. Electrochemical Tests of Composite Plating Bath

The electrochemical tests were performed using the instruments and chemicals shown in Table 3.

**Figure 4:**
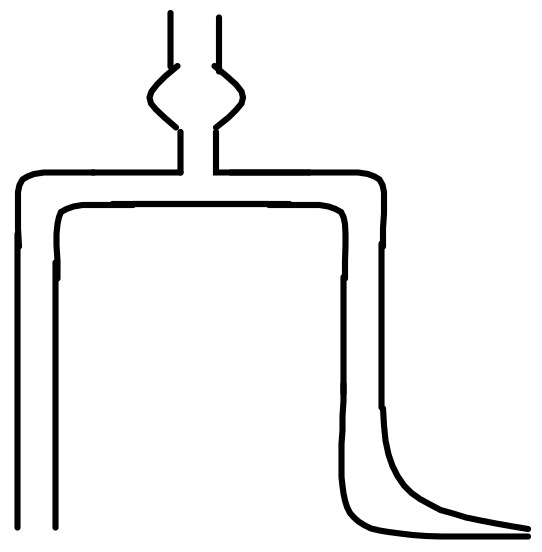
Schematic diagram of the Luggin capillary salt bridge.

**Figure 5:**
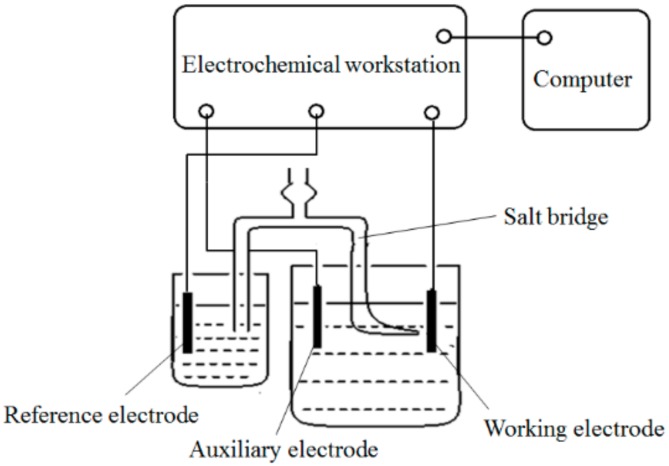
Diagram of the electrochemical test circuit and system.

The electrochemical behavior of Ni^2+^ ion was studied by testing the polarization curve, using the linear sweep voltammetry technique. Measurements were performed by a CHI660B electrochemical working station, Beijing Huake Putian Science and Technology Co., Ltd., Beijing, China. A standard three-electrode cell was used for the electrochemical measurement, which consisted of a Q235A steel plate as the working electrode, a nickel plate as the auxiliary electrode, and a saturated calomel electrode (SCE) as the reference electrode. The SCE was put in a little beaker containing saturated potassium chloride solution, and a Luggin capillary salt bridge was used to connect the SCE to the bath. Schematic diagrams of the Luggin capillary salt bridge and the three-electrode system are shown in Figure 4 and Figure 5, respectively.

The polarization curves were recorded at scanning rates of 0.5 mV/s, and in the potential range of open-circuit potential of about −1.5 V. Effects of nanodiamond concentration on the polarization process were investigated in a bath containing nanodiamond concentrations of 4, 8, 12, and 16 g/L, with the stirring rate fixed at 10 r/min. The influence of stirring rate on the polarization process was studied under stirring speeds of 2.5 r/min, 5 r/min, 7.5 r/min, and 10 r/min, separately, with other conditions unchanged and a nanodiamond concentration of 16 g/L in the bath.

### 2.5. Characterization

The morphology of the nanodiamond particles was measured with a transmission electron microscope (TEM; H-8100, Hitachi, Tokyo, Japan). The X-ray diffraction (XRD) patterns of the nanodiamond were recorded with a diffractometer (RAX-10, Hitachi, Tokyo, Japan). The elastic modulus and hardness of the composite coatings were determined by the nano-indenter XP tester (NANOVEA, Irvine, CA, USA), the loading rate was the same as the unloading rate at 40 nm/s, the pressed depth was 2 µm, and the pressure was maintained for 10 s at the maximum pressed depth. As the thickness of the coating was about 20 µm and the pressed depth was 10% of the thickness of the coating, the mechanical properties of the coating would not be affected by the substrate. The surface roughness value *Ra* was measured with a 2302A surface profile measuring instrument (Harbin Measuring and Cutting Tool Group Co., Ltd., Harbin, China), and the measurement parameters were as follows: Sampling length 0.8 mm, evaluation length 4 mm, and driving box gliding speed 0.5 mm/s. The surface morphology of the coating was analyzed with the X650 scanning electron microscope (SEM, Hitachi, Tokyo, Japan), and the grain distribution situation was observed with a voltage of 10 kV and the voltage magnified by 5000 to 10,000 times.

## 3. Results and Discussion

### 3.1. Nanodiamond Test

**Figure 6:**
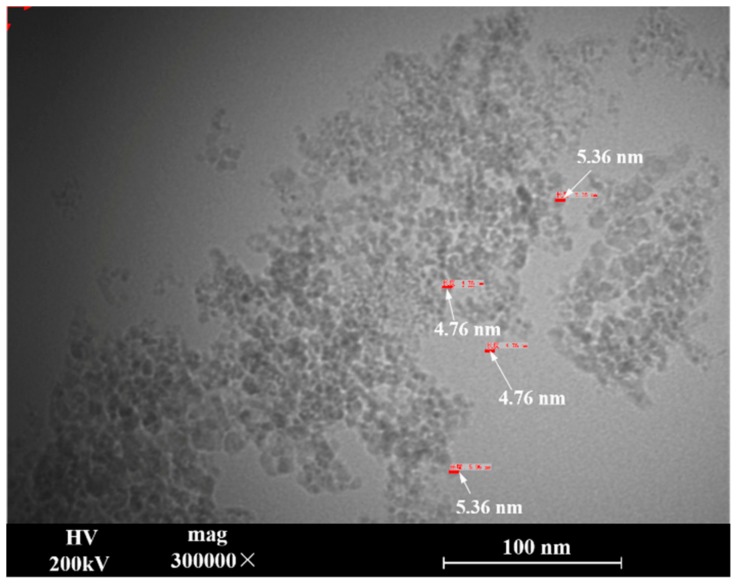
Transmission electron microscopy images of nanodiamond powder (300,000×).

Figure 6 shows transmission electron microscopy photographs of the purified nanodiamond in the ultradispersed state, with magnification of 300,000 times. The particle size distribution was found to range from 4.8 nm to 5.4 nm. The main components were carbon 88–93%, water ≤ 3%, N ≤ 2.5%, and O ≤ 10%. The contents of the impurity elements (gas elements excluded) in the sample were determined by atomic emission spectroscopy and are listed in Table 4.

All of the purified nanodiamonds measured by X-ray diffraction showed a cubic diamond phase, but the relative strengths of the five common diffraction peaks were very different compared to conventional diamond grains. The specific data are shown in Table 5.

**Figure 7:**
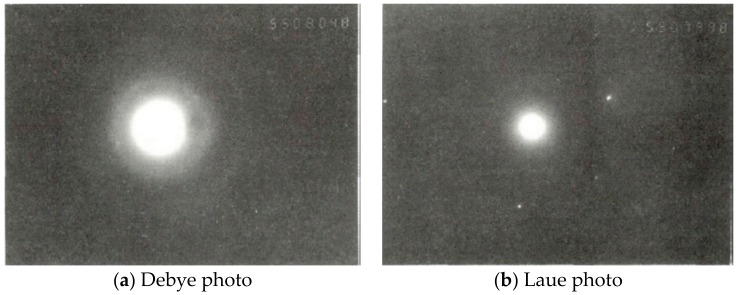
X-ray diffraction picture of nanodiamond grain.

Figure 7 shows the Debye and Laue photos of nanodiamonds. Based on this, the interplanar spacing of the nanodiamonds could be calculated. It was found that the interplanar spacing of the nanodiamond crystal faces (111) increased from 0.206 nm to 0.207 nm, and the interplanar spacing of the crystal plane (220) was reduced from 0.1261 nm to 0.1231 nm, which indicates that the crystal lattice of the nanodiamond had a significant distortion.

### 3.2. Electrochemical Test

#### 3.2.1. Anodic Polarization Process of Nickel Plate

**Figure 8:**
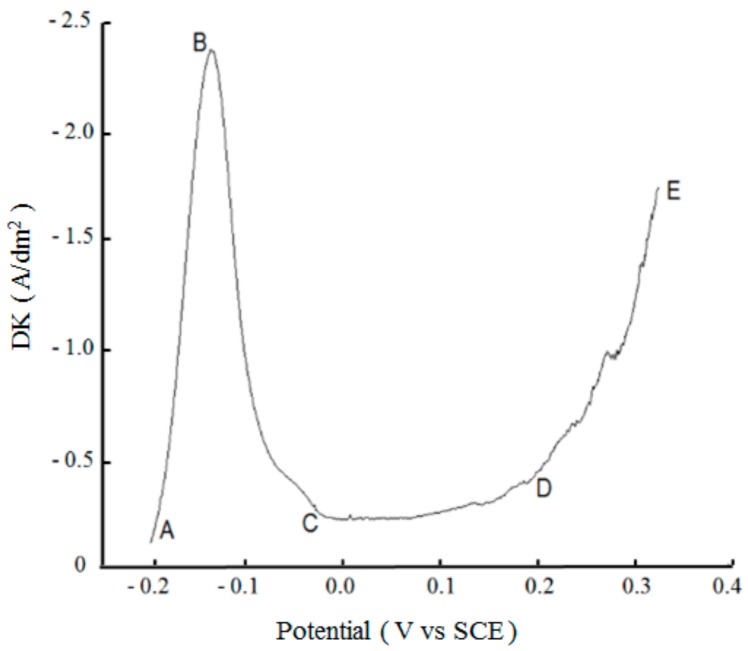
Anodic polarization curve of the nickel anode.

Figure 8 shows the anodic polarization curve of the nickel plate measured in the potential range of 0 to −0.35 V. The polarization curve shows four obvious segments with the following characteristics: AB segment: Within the corresponding potential range, the anode was dissolved normally and the surface was in the active state, known as the normal dissolution zone; BC segment: The anode metal surface changed from active to passive state, known as the activation-passivation transition zone; CD segment: The metal dissolution rate was reduced to a minimum and basically had no change in the potential, and this segment was similar to a horizontal line, known as the stably passive zone; DE segment: The anode polarization continued to increase and the anode current increased again, which is known as the transpassive phenomenon, and this segment is called the transpassive zone. The transpassive phenomenon is due to the generation of high-valence metal ions or the presence of other anodic reactions. According to the results in Figure 8, the current density of the nickel anode cannot exceed 2.4 A/dm^2^ to ensure normal dissolution of the nickel anode. Based on the determined anodic current density value, the nickel anodic area was determined to be 4.2 cm^2^ during the plating.

#### 3.2.2. Effects of Nanodiamond Concentration on Cathodic Polarization Curves

**Figure 9:**
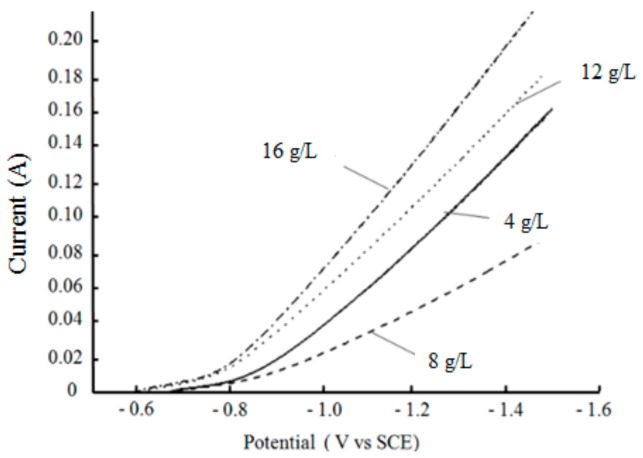
Cathodic polarization curves tested in solutions containing different concentrations of nanodiamond powder.

Figure 9 shows the cathodic polarization curves measured in the bath, with different nanodiamond concentrations. It can be seen that the current was low when the potential was in the range of −0.56 to −0.8 V, which indicates that the remarkable reduction of Ni^2+^ did not yet start and therefore the current was small. When the potential was lower than −0.8 V, the reduction of Ni^2+^ started to accelerate and the current increased significantly. Comparing the over-potential of the different curves under the same current, it was found that the over-potential first increased and then decreased with increased nanodiamond concentration, and the highest over-potential was obtained when the nanodiamond concentration was 8 g/L. These results may be due to the adsorption and deposition of nanodiamond particles on the surface of the electrode having a certain hindering effect on the discharge of metal ions, which is favorable for the deposition of nanodiamond particles in the coating. However, when the nanodiamond content in the bath is too high, the hindering effect weakens and the reduction rate of Ni^2+^ is accelerated, which leads to a decreased nanodiamond quantity in the composite coating.

#### 3.2.3. Effect of Stirring Speed on Cathodic Polarization Curves

**Figure 10:**
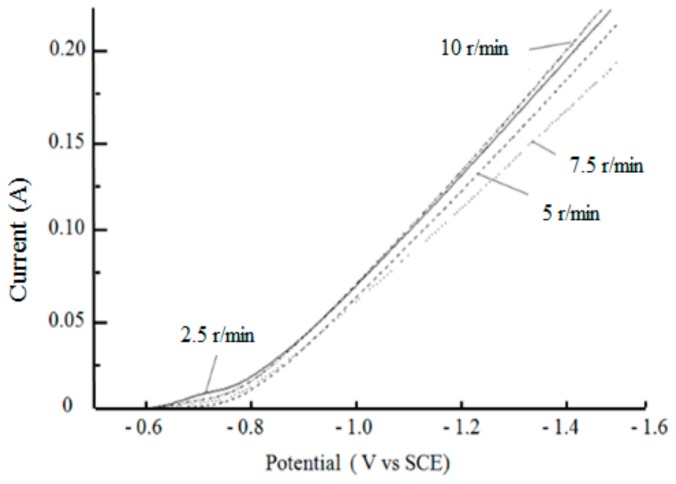
Cathodic polarization curves tested under different stirring speeds.

Figure 10 shows the cathodic polarization curves measured at different stirring speeds with the fixed nanodiamond concentration of 16 g/L in the bath. Comparing the over-potential of the different curves under the same current, it can be seen that the over-potential first increased and then decreased with the increased stirring speed, and the maximum over-potential was obtained when the stirring speed was 7.5 r/min. Stirring can facilitate the diffusion of nickel ions in the bath, and also improve the bath dispersion. By stirring, the consumed nickel ions near the cathode can be quickly replenished, so as to reduce the concentration polarization and improve current efficiency. Simultaneously, stirring can also allow the hydrogen separated out during the electroplating to escape and reduce pinholes and pock marks on the surface of the coating [22,23]. When the stirring speed is low, the nanodiamond particles in the plating solution are not in a well-suspended state, and the sedimentation will reduce the content of nanodiamond in the coating. On the other hand, when the stirring speed is too high, the particles are in vigorous motion and the impact on the cathode surface is too large, while the residence time of nanodiamond particles on the cathode surface is too short. So it is difficult for the particles to stay on the cathode surface and be further caught in the coating. Therefore, an extremely high stirring speed is not conducive for the preparation of Ni/nanodiamond composite coatings.

### 3.3. Effect of Nanodiamond Concentration in Bath on Properties of Composite Coatings Prepared by DC Electroplating

**Figure 11:**
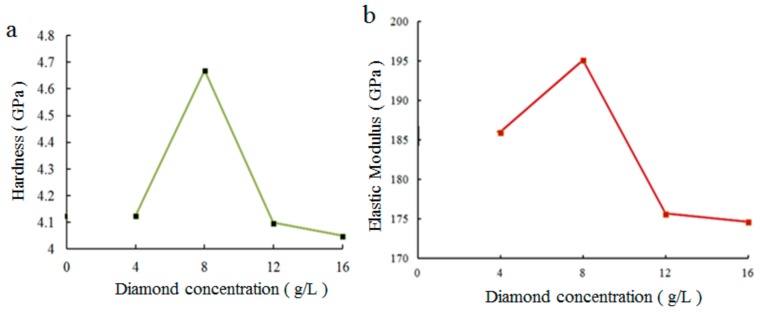
Effect of nanodiamond concentration on mechanical properties of composite coatings: (**a**) Hardness, and (**b**) elastic modulus.

The hardness and elastic modulus of the as-prepared coatings were tested, and the results are shown in Figure 11. It can be seen that the hardness and elastic modulus of the composite coating increased initially, and then decreased with increased nanodiamond content in the plating solution. When the content of nanodiamonds in the bath was 8 g/L, the hardness and elastic modulus of the composite coating reached the maximum values of 4.66 GPa and 195.1 GPa, respectively. When the content was 16 g/L, the hardness and elastic modulus were at the minimum values.

The range of coating surface roughness parameter *Ra* was 1.19–1.35 µm. When the nanodiamond concentration was 8 g/L, *Ra* reached the minimum value of 1.19 µm, and when the concentration was 16 g/L, *Ra* reached the maximum value of 1.35 µm.

**Figure 12:**
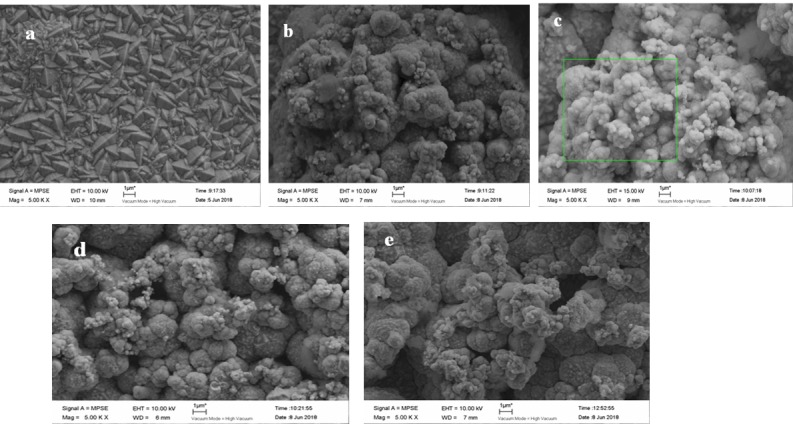
SEM images of composite coating plated at nanodiamond concentrations from 0 g/L to 16.0 g/L in plating solution: (**a**) 0 g/L, (**b**) 4.0 g/L, (**c**) 8.0 g/L, (**d**) 12.0 g/L, and (**e**) 16.0 g/L.

Figure 12 presents the surface morphology of the coating, with various nanodiamond concentrations ranging from 0 g/L to 16 g/L in the plating solution. It is shown that with the addition of nanodiamonds, the crystal shape of the composite coating changed from a rhombus to a spherical morphology. It can be observed that the surface morphology of the composite coating was relatively dense and the grain was compact when the nanodiamonds were contained in the plating solution. As the nanodiamond concentration in the plating solution increased, the content of nanodiamonds in the composite coating also increased. The smaller the surface grain size, the more significantly uniform the distribution, because the dispersion of nanodiamonds in the matrix promotes nucleation, effectively preventing grain growth and refining the surface. Guglielmi [24] suggested that the co-deposition of particles was mainly achieved through two successive steps, weak adsorption and strong adsorption, and then the particles can be embedded in the composite coating. According to such an adsorption mechanism, with the increased content of nanodiamond particles in the bath, the number of particles entering the weak adsorption process will increase due to stirring and diffusion, so the particles embedded in the coating will increase, too. However, when the nanodiamond content in the bath is more than 8 g/L, the hardness and elastic modulus of the composite coating are reduced. The main reasons are as follows: Firstly, when the content of nanodiamond particles in the plating solution is too high, there will be a shielding effect between the particles, which will reduce the probability that particles will enter the coating. Moreover, as the nanodiamond particles flow in the plating solution, the probability that they will collide increases, which would cause the flowing nanodiamond particles to scour the cathode surface. Thus, the number of particles embedded in the coating and the mechanical properties of the coating are both reduced. Secondly, due to the special properties of nanoparticles, an extremely high content of nanodiamond particles in the bath will lead to their accelerated agglomeration, so that the dispersion of the particles will decrease and sedimentation will occur in the plating solution. The increased particle size will not only slow down the transmission speed, but also increase the time for the nickel ions to cover or bury the diamond particles—i.e., the adsorption and deposition of nanodiamond particles on the electrode surface will have a certain hindering effect on the discharge of nickel ions.

### 3.4. Effect of Stirring Speed on Properties of Composite Coatings Prepared by DC Electroplating

Based on the above experimental results, a comparison experiment was carried out with nanodiamond contents of 8 g/L and 16 g/L in the bath, to study the effect of stirring speed during electroplating on the properties of the resulting composite coating.

**Figure 13:**
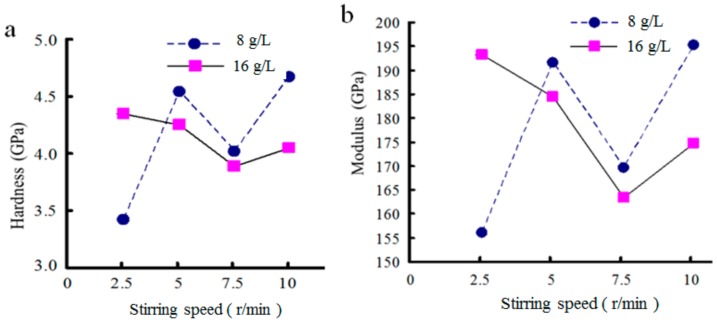
Effects of stirring speed on mechanical properties of composite coatings: (**a**) Hardness, and (**b**) elastic modulus.

Figure 13 shows the effects of stirring speed on the hardness and elastic modulus of the composite coating. It can be seen from the figure that when the nanodiamond concentration in the plating solution was 16 g/L and the stirring speed was 2.5 r/min, and when the nanodiamond concentration was 8 g/L and the stirring speed was 10 r/min, the hardness and elastic modulus of the composite coating reached maximum values.

**Figure 14:**
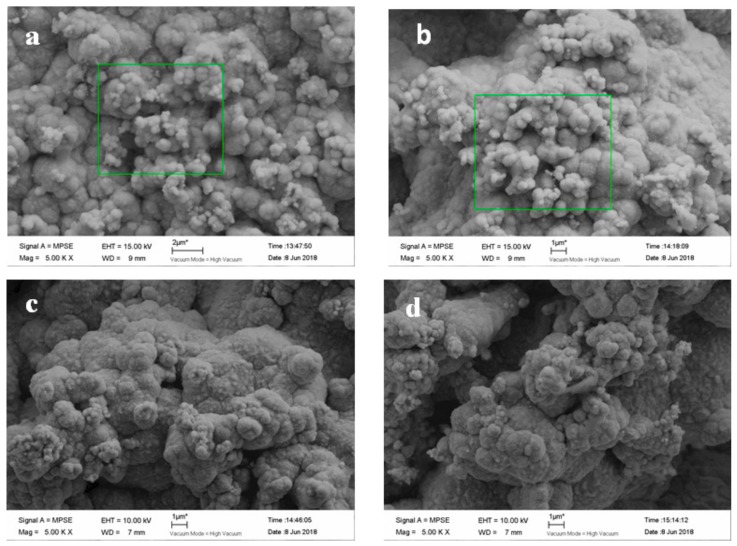
SEM images of composite coating obtained by electroplating at different stirring speeds: (**a**) 10 r/min, (**b**) 7.5 r/min, (**c**) 5 r/min, and (**d**) 2.5 r/min.

Figure 14 shows the surface morphology of the composite coating electroplated at different stirring speeds and with a nanodiamond concentration of 8 g/L in the bath. It can be seen from the figure that the surface of the composite coating prepared at the stirring speed of 10 r/min is relatively uniform and smooth with fine crystallization, but those prepared at other stirring speeds are relatively rough. The surface morphology of the composite coating is consistent with the obtained results of surface roughness. When the nanodiamond concentration was 8 g/L, *Ra* reached the minimum value of 1.19 µm.

### 3.5. Effect of Electroplating Mode on Properties of Composite Coatings

In order to study the effect of the electroplating mode on the properties of the composite coating, tests were carried out by changing the electroplating mode while keeping the other process parameters unchanged. The composite coatings were separately prepared by double-pulse electroplating and DC electroplating under two conditions: With a double-pulse positive operating time of 50 and 100 ms, respectively. Under these conditions, the hardness, elastic modulus, surface roughness, and surface morphology of the as-prepared composite coatings were investigated, as described below.

#### 3.5.1. Effect on the Hardness and Elastic Modulus of Composite Coatings

Table 6 lists the measurement results of the hardness and elastic modulus of the composite coatings prepared by different electroplating modes. Under the same conditions, the hardness and elastic modulus of the composite coating prepared by the double-pulse electroplating mode were higher than those of the composite coating prepared by the DC electroplating mode. Moreover, the hardness and elastic modulus of the composite coatings with a positive pulse operating time of 50 ms were better than those of the coating prepared with a positive pulse operating time of 100 ms.

#### 3.5.2. Effect on Surface Roughness Parameter *Ra* of Composite Coatings

Table 7 shows the measurement results of the surface roughness parameter *Ra* for the composite coating prepared by different electroplating modes. It can be seen that *Ra* of the composite coating prepared by the pulse electroplating mode is smaller than that of the composite coating prepared by the DC electroplating mode. According to the experimental results in Table 7, the composite coating prepared with a positive pulse operating time of 50 ms has a smaller surface roughness parameter *Ra*, and its coating surface is relatively flat and smooth with better wear resistance.

#### 3.5.3. Effect on Surface Morphology of Composite Coatings

**Figure 15:**
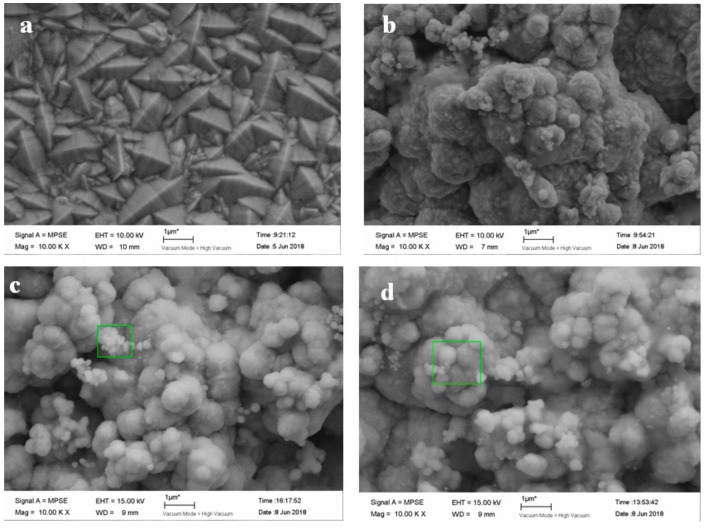
SEM images of coating prepared by different electroplating modes: (**a**) DC electroplating Ni coating, (**b**) single-pulse plating, (**c**) double-pulse electroplating with positive pulse operating time of 50 ms, and (**d**) double-pulse electroplating with positive pulse operating time of 100 ms.

Figure 15 shows SEM images of the composite coatings prepared under different electroplating modes. It can be seen that the surface particle size of the composite coating prepared by DC electroplating is larger than that prepared by pulse electroplating, and the surface of the composite coating is not flat. In the pulse electroplating process, the formation of a new crystal nucleus dominates and the growth of the crystal nucleus is the secondary process [25]. As a result, the formed deposition layer is relatively flat, the composite coating is smoother, and the crystal is the finest when the positive pulse operating time is 50 ms (Figure 15c). The experimental results show that the performance of the composite coating prepared by the double-pulse electroplating mode is much better than that prepared by a single pulse, a single pulse is much better than DC electroplating, and a positive pulse operating time of 50 ms provides better performance of the composite coating than the operating time of 100 ms.

This occurred due to the following effects exerted by the nanodiamonds on the composite coating: (1) Dispersion strengthening effect: When the nanodiamonds were distributed in the nickel matrix, the nickel matrix bore the load under the external force and the nanodiamonds could effectively prevent the dislocation movement of nickel and microcrack growth; (2) the refined crystalline strengthening effect: Where the nanodiamonds were adsorbed onto the matrix metal surface and embedded within—forming a point effect—for better deposition of nickel atoms, and the nucleation rate exceeded the grain growth rate and the grain size was reduced, enhancing the mechanical properties of the composite coating; and (3) the dislocation strengthening effect: Due to the embedding of the nanodiamonds, the lattice defects—including the stacking fault and twin boundaries—were higher in the composite coating and the crystals near the grain boundary were distorted, and consequently the grain movement within the composite coating was inhibited and the corresponding performance was improved [24].

## 4. Conclusions

The effects of nanodiamond concentration on the electrochemical behavior of Ni^2+^ ion was first investigated by measuring the polarization curves in a bath with different nanodiamond concentrations to determine the optimum coating conditions. Then, the effects of DC and double-pulse electroplating modes on the surface roughness, hardness, and elastic modulus of the deposited Ni/nanodiamond composite coating on Q235A substrate were further studied. The following conclusions were drawn: The nanodiamond concentration in the bath affected the reduction of over-potential of Ni^2+^ ion and reached the maximum value at a concentration of 8 g/L. The hardness and elastic modulus of the Ni/nanodiamond composite coating prepared by the DC electroplating mode reached the maximum values at 4.68 GPa and 194.30 GPa, respectively, at a nanodiamond concentration of 8 g/L in the bath. In the DC electroplating process, the effect of stirring speed on the hardness and elastic modulus of the composite coating was related to the nanodiamond concentration in the bath. When the concentration was 8 g/L and the stirring speed was 10 r/min, the hardness and elastic modulus reached their maximum values. Under the same process conditions, the surface of the composite coating prepared by the pulse electroplating mode was smoother, and the mechanical properties were better than that prepared by the DC electroplating mode, of which the double-pulse electroplating mode was best. A positive pulse operating time of 50 ms provided better performance of the composite coating than an operating time of 100 ms. The findings of this work are expected to help guide future practical applications of nanodiamond composite coatings.

## Figures and Tables

**Table 1:** Solution formulas and process parameters of the degreasing treatment.

Chemicals and Parameters	Chemical Degreasing	Electrochemical Degreasing
NaOH (g/L)	65	15
Na_2_CO_3_ (g/L)	17.5	55
Na_3_PO_4_·12H_2_O (g/L)	17.5	35
Na_2_SiO_3_·9H_2_O (g/L)	5	7.5
Temperature (°C)	70	70
Time (min)	2	2
Current (A)	–	0.2

**Table 2:** Double-pulse electroplating process parameters.

Parameter	Experiment 1		Experiment 2	
	Positive	Negative	Positive	Negative
Pulse frequency (Hz)	1000	1000	1000	1000
Average current (A)	0.06	0.02	0.06	0.01
Operating time (ms)	50	10	100	10
Duty cycle (%)	20	10	20	10

**Table 3:** Instruments used for electrochemical testing.

Instrument	Model	Manufacturer
Electrochemical workstation	CHI660E	Beijing Huake Putian Science and Technology Co., Ltd. (Beijing, China)
Heat-collection thermostatic heating magnetic stirrer	DF-101S	Gongyi City Yuhua Instrument Co., Ltd. (Gongyi, China)
Working electrode (steel disc)	Q235A	–
Auxiliary electrode (nickel plate)	–	–
Reference electrode (saturated calomel electrode, SCE)	CHI150	Tianjin Aida Hengsheng Technology Development Co., Ltd. (Tianjin, China)
Luggin capillary salt bridge	–	Self-made

**Table 4:** Contents of impurity elements in ultradispersed nanodiamond powder (% by mass).

**Element**	**Fe**	**Cr**	**Si**	**Al**	**Na**	**K**	**Cu**
Content	0.150	0.070	0.300	0.005	0.030	0.002	0.005
**Element**	**Ca**	**Mg**	**Mn**	**Ti**	**Pb**	**Incombustible Substance**	**Total**
Content	0.002	0.005	0.001	0.010	0.001	0.95	1.531

**Table 5:** Relative intensity of diffraction peaks in XRD spectra.

Crystal Face	(111)	(220)	(113)	(400)	(331)
Nanodiamond Standard diamond	100 100	16 25	0.6 16	0.4 8	0.2 16

**Table 6:** Hardness and elastic modulus of composite coatings prepared by different electroplating modes.

Electroplating Mode			Hardness (GPa)	Elastic Modulus (GPa)
DC electroplating			4.68	194.30
Double-pulse electroplating	Operating time (ms)	50	5.22	197.38
		100	5.14	192.5

**Table 7:** Surface roughness parameter *Ra* of composite coatings prepared by different electroplating modes.

Electrodeposition Mode			Surface Roughness *Ra* (µm)
DC electroplating			1.184
Double-pulse electroplating	Operating time (ms)	50	0.792
		100	1.006

## References

[1] Huang W., Zhao Y., Wang X. (2013). Preparing a high-particle-content Ni/diamond composite coating with strong abrasive ability. Surf. Coat. Technol..

[2] Vaezi M.R., Sadrnezhaad S.K., Nikzad L. (2008). Electrodeposition of Ni–SiC nano-composite coatings and evaluation of wear and corrosion resistance and electroplating characteristics. Colloids Surf. A Physicochem. Eng. Asp..

[3] Walsh F.C., Low C.T., Bello J.O. (2015). Influence of Surfactants on Electrodeposition of a Ni-Nanoparticulate SiC Composite Coating. Trans. Inst. Mater. Finish..

[4] Low C.T.J., Wills R.G.A., Walsh F.C. (2006). Electrodeposition of composite coatings containing nanoparticles in a metal deposit. Surf. Coat. Technol..

[5] Shrestha N.K., Takebe T., Saji T. (2006). Effect of particle size on the co-deposition of diamond with nickel in presence of a redox-active surfactant and mechanical property of the coatings. Diam. Relat. Mater..

[6] Grosjean A., Rezrazi M., Takadoum J., Bercot P. (2001). Hardness, friction and wear characteristics of nickel-SiC electroless composite deposits. Surf. Coat. Technol..

[7] Hamed M., Saeed A. (2012). Deposition characterization and electrochemical evaluation of Ni-P-nano diamond composite coatings. Appl. Surf. Sci..

[8] Jappes J.W., Ramamoorthy B., Nair P.K. (2009). Novel approaches on the study of wear performance of electroless Ni-P/diamond composite deposites. J. Mater. Process. Technol..

[9] Wun-Hsing L., Sen-Cheh T., Kung-Cheng C. (1999). Effects of direct current and pulse–plating on the co-deposition of nickel and nanometer diamond powder. Surf. Coat. Technol..

[10] Luan X.W., Wang M.Z., Zhao Y.C. (2005). Study on electroplated nickel-nanodiamond composite coating. Diam. Abras. Eng..

[11] Petrov I., Detkov P., Drovosekov A., Ivanov M.V., Tyler T., Shenderova O., Voznecova N.P., Toporov Y.P., Schulz D. (2006). Nickel galvanic coatings co-deposited with fractions of detonation nanodiamond. Diam. Relat. Mater..

[12] Wang L., Gao Y., Liu H., Xue Q., Xu T. (2005). Effects of bivalent Co ion on the co-deposition of nickel and nano-diamond particles. Surf. Coat. Technol..

[13] Medeliené V., Stankevič V., Bikulčius G. (2003). The influence of artificial diamond additions on the formation and properties of an electroplated copper metal matrix coating. Surf. Coat. Technol..

[14] Mandich N.V., Dennis J.K. (2001). Codeposition of nanodiamonds with chromioum. Plat. Surf. Finish..

[15] Wang L.P., Gao Y., Xue Q.J., Liu H.W., Xu T. (2004). Effect of nano-diamond particulates on the microstructure and wear-resistance of electrodeposited Ni-matrix coatings. Tribology.

[16] Xie H.B., Zhang L.X., Ma X.K. (2006). A study on properties and composite electroplating process of Cr-nanodiamond. Mater. Protect..

[17] Wang B.C., Zhu Y.W., Xu X.Y., Xie S.Z. (2004). Preparation of Nanodiamond Suspension for Composite Cr-nanodiamond Coating. Surf. Technol..

[18] Yang D., Zhang H., Tong X., Zhang Z., Zheng Z., Fan D. (2002). Tribological Bahavior of Chromium Based Nano-diamond composite coating. Heat Treat. Met..

[19] Zhao Y., Liu M.H., Feng L., Meng Y., Li F., Chen Z. (2014). The process optimization for Ni coating electrodeposited on Q235A. Mater. Protect..

[20] Yuan X., Wang Y., Sun D., Yu H. (2010). Influence of pulse parameters on the microstructure and microhardness of nickel electrodeposits. Mater. Sci. Technol..

[21] Feng L.M., Wang Y. (2010). Electroplating Technology.

[22] Liu H.J., Shi X.P. (2006). On research of HT-I reagent for controlling pinpoint hole roof when nickel plating. Tianjin Chem. Ind..

[23] Luo H.X., Huang X.G., Mei Y.X. (2014). Causes and solutions of pinholes fault on the Nickel coating. Hydraul. Pneum. Seals.

[24] Guglielmi N. (1972). Kinetics of the deposition of inert particles from electrolytic baths. J. Electrochem. Soc..

[25] Xiao X., Chu R.B. (2004). Causes and trouble–shootings for pores and pits on nickel electrode posits. Electroplat. Finish..

